# Factors Influencing Patients’ Choice of Public or Private Hospitals in Riyadh City: A Cross-Sectional Study

**DOI:** 10.7759/cureus.78756

**Published:** 2025-02-09

**Authors:** Nada M Almosa, Wadi Alonazi

**Affiliations:** 1 Risk Management, King Saud University, Riyadh, SAU; 2 Health Adminstration, King Saud University, Riyadh, SAU

**Keywords:** healthcare costs, health services, hospital selection, patient choice, private and public hospitals

## Abstract

Background and aim: To address healthcare challenges in Saudi Arabia, such as quality, efficiency, and increasing healthcare demands, the healthcare sector is undergoing significant transformation under the Healthcare Transformation Program in both public and private sectors, aligned with Vision 2030 for the country. The aim of this study was to investigate the factors influencing the preference of patients between public and private hospitals in Riyadh, Saudi Arabia.

Methods: This was a descriptive cross-sectional study. Primary data was collected using a 38-item self-administered questionnaire. The questionnaire included demographic data and patient perceptions of services, efficiency, cost, and hospital selection. The data was collected using convenience sampling from 600 participants living in Riyadh who have attended public or private hospitals. Reliability was evaluated using Cronbach’s alpha (α=0.869), demonstrating strong internal consistency. Item-total correlations across domains were statistically significant (p<0.01), supporting validity. Statistical analyses, including descriptive statistics and an independent sample t-test, were performed.

Results: The majority of participants were Saudi nationals (577, 96.2%) and female (399, 66.5%). Participants' ages ranged from 20 to 40 years. More than half of the respondents (303, 50.5%) held a bachelor's degree, while 40.2% reported a monthly family income of 10,000-20,000 SR. Hospital selection (3.79±0.65) was the strongest factor influencing patient preferences. Services provided (2.68±0.65) and efficiency (3.06±0.67) received neutral responses, while the cost of treatment (2.56±0.66) was not a significant factor. Education significantly influenced preferences, with highly educated patients favoring private hospitals. Also, non-Saudi patients valued private hospitals for better cost-effectiveness and services.

Conclusions: Private hospitals are perceived as superior by people living in Riyadh compared to public hospitals when it comes to service level and efficiency. Respondents did not feel that the indirect costs of using public hospitals were significant. It shows the lower cost of public hospitals remains an important attraction that encourages users to rely on public hospitals. Nevertheless, respondents justify the higher cost of private hospitals because of the superior service level. Availability of modern equipment in the hospital, the reputation of doctors employed by the hospitals, overall appearance and outlay of the hospital facilities, hygiene, and location are also important factors that influence their choice of the hospital.

## Introduction

Healthcare plays a vital role in improving life expectancy and the standard of living, making the healthcare industry in Saudi Arabia essential. Its importance will continue to grow as the average age of the population rises over time [1]. The Health Sector Transformation Program, based on the Kingdom’s Vision 2030, aims to address the challenges faced by the healthcare sector in Saudi Arabia. Some of these challenges include an attempt to boost quality, efficiency, effectiveness, and the level of protection provided by the healthcare sector against health-related risks experienced by residents in Saudi Arabia [2]. Public hospitals, including ministry of health hospitals, military hospitals, and referral hospitals, have been the main providers of healthcare services. However, private hospitals are also playing an important role in serving the health-related needs [3]. Researchers have taken an increased interest in understanding any potential difference in service quality and provision of the service from public versus private hospitals [4]. This is due to the implications of these differences in service quality on the preferences of patients and the future demand for services provided by private and public hospitals in Saudi Arabia. 

Even though various studies have been undertaken to understand the preference of patients between public and private hospitals in Saudi Arabia, no single study has combined the relevant to undertake a quantitative study to investigate the perception of patients, which influences their choice between public and private hospitals. The key factors mentioned in the literature include services, efficiency, cost of treatment, and hospital selection [1,5-7]. It presents a gap in the existing literature, which is to be addressed through this paper. The lack of existing research on these factors in a single paper highlights the limited existing research that has examined the factors influencing the preference of patients between public and private hospitals in Saudi Arabia. Furthermore, work on this topic is also limited when it comes to Riyadh, a major Saudi Arabian city and its capital. Given the growing budget allocated to health care and an increase in spending on patient care over time, the topic of factors that impact patient preference between public and private hospitals in Riyadh is of practical significance.

Four key factors reported in the literature that affect the patient preference in selecting between public and private hospitals are service quality, efficiency, cost, and hospital selection. Private hospitals tend to perform better on dimensions of the SERVQUAL framework, as concluded by Alumran et al. [1]. These dimensions include tangibles, reliability, responsiveness, empathy, and assurance. Private hospitals have more visually appealing medical and healthcare equipment (tangibles) and are more likely to provide a reliable service (i.e., when a hospital promised to fulfill a certain objective in a particular time frame, a private hospital was more likely to achieve that). Employees in the private sector were more professional, provided a differentiated and personalized service, were never too busy to take the time out and respond to requests from patients and their family members, and were more likely to provide individualized attention [1]. Thus, patients perceive private hospitals in Saudi Arabia to possess superior facilities and quality of service. With the growing pressure on public sector funding and the drive towards privatization of the healthcare industry in Saudi Arabia, public hospitals have failed to keep up with investment in the latest medical equipment and associated technology, as undertaken by private hospitals [8,9].

The organizational objective also explains the differences in the level of services offered by private and public hospitals. Private hospitals are incentivized to invest in infrastructure to improve their service level, given profit maximization as their primary objective. Private hospitals are profit-oriented organizations, unlike the non-profit nature of public sector hospitals [10,11]. Over time, greater investment in private hospitals relative to public hospitals is also reflected in the level of cleanliness and hygiene, as well as the quality of food provided [12]. It explains why patients perceive private hospitals to provide a higher quality of service and possess superior facilities compared to public hospitals.

When it comes to efficiency, public hospitals based on non-profit motives do not have the same incentives and drive, especially in the short run [7]. The increased inherent efficiency of the private hospitals relative to the public hospitals means there is a strong economic rationale for promoting the privatization of the healthcare sector, as outlined by the Health Sector Transformation Program based on the Kingdom’s Vision 2030 to address the challenges faced by the healthcare sector in Saudi Arabia [13]. 

Privatization is also preferred because, despite the significant resources (government spending) allocated to healthcare, public hospitals continue to be under strain because of challenges such as population growth, aging population, obesity, and an increase in sedentary lifestyles contributing to greater demand for healthcare services in Saudi Arabia [14]. By their very nature, hospitals in the private sector are driven by the need to improve their profit because enterprises in the private sector focus on profit maximization as their primary objective, which in turn drives their emphasis on operational efficiency, customer focus, and other secondary objectives [15,16]. These arguments help to explain why private sector hospitals in Saudi Arabia are more efficient: even though profit remains their primary motive, the emphasis on maximizing profitability in the private sector (unlike public sector hospitals) provides a strong incentive to improve efficiency with which the private sector hospitals are run.

The cost incurred on treatment is another variable that influences patient preference in selecting between public and private hospitals, which are service quality, efficiency, cost, and hospital selection. The increased indirect cost of healthcare in public hospitals in Saudi Arabia is an important factor that incentivizes the growing number of patients who seek healthcare from private hospitals. Within private hospitals, even though the patients have to pay the direct cost of healthcare, the inelastic demand for certain critical medical conditions means patients have a preference for quality of service over the cost incurred [16]. Thus, patients appear to have a preference for private hospitals because of certainty about the quality and timeliness of the treatment they will receive. Finally, hospital selection as a variable involves consideration of the quality of healthcare, availability of advanced medical equipment and technology due to its influence on the quality of healthcare, and the reputation of the hospital staff as significant in influencing their choice of the hospital [1].

This study aimed to investigate the factors influencing the preference of patients between public and private hospitals in Riyadh, Saudi Arabia. To fulfill this aim, the study focuses on investigating patients’ perception of services provided in public and private hospitals, analyzing the perception amongst patients relating to the efficiency of public and private hospitals, evaluating the perception of patients around the cost of treatment within public and private hospitals, and review the patients’ perception of the factors influencing the choice of public and private hospitals.

## Materials and methods

Study design and population

This is a descriptive cross-sectional study that investigated the factors influencing citizens and residents about their preferences in choosing between public and private hospitals in Riyadh, Saudi Arabia. The study was conducted with a well-defined questionnaire; an initial pilot survey was conducted to examine the questionnaire on the basis of convenience sampling. The population relevant to the study is all adult residents who have previously attended the public and private hospitals in Riyadh. The questionnaire was available to participants during the period from September 2022 to September 2023. The ethical approval for this study was obtained from the Institutional Review Board (IRB) of King Saud University (approval number: KSU-HE-22-438).

Convenience sampling is a method adopted by researchers to collect data from the research participants that is easily accessible [17]. There are several reasons why the convenience sampling technique was chosen in this study. Firstly, as there is a large population (all adult citizens and residents who have previously experienced public and private hospitals in Riyadh), convenience sampling is a time-efficient and cost-effective way to reach many respondents [18]. Secondly, convenience sampling is a straightforward sampling method because there are fewer rules to follow, which reduces complexity in data collection [19]. Convenience sampling was effective in helping to increase the sample size. The survey was distributed electronically through the link to Google form survey via social media platforms. By using the convenience sampling technique, 600 survey responses were received.

Variables

A 38-item self-administered questionnaire was used, comprising six parts. The first part included the research title and informed consent obtained from the participants with the option to respond to the survey in either English or Arabic language. The second part of the survey focused on the demographic characteristics of the respondents, such as nationality, gender, age, qualification, employment status, marital status, family monthly income, and number of members in the family. The third part consisted of 13 statements requiring the respondents to provide their viewpoints on how they perceive the services to be in private and public hospitals. The fourth part focused on patient perception regarding efficiency, which included nine statements on which responses were obtained. This was followed by the fifth part, which investigated patient perception regarding the cost of treatment in public and private hospitals. Finally, the survey concentrated on understanding the perception of respondents with respect to the choice of hospital. 

Statistical analysis

Reliability and validity were addressed, followed by the use of descriptive statistics and independent sample t-tests. The internal consistency of the study questionnaire was assessed by Cronbach's alpha coefficient for the responses of all subjects. Table 1 shows that all item-total correlation coefficients for the domain's items were statistically significant at the 1% level, indicating strong internal consistency between each item and the total score of its respective domain. Additionally, Table 2 demonstrates that all four domains were significantly correlated with the overall tool.

**Table 1:** Item-total correlation for the study domains. ** correlation is significant at the level 0.01.

Study domain	Item-total correlation
Services/facilities	
Item 1	0.653**
Item 2	0.461**
Item 3	0.638**
Item 4	0.591**
Item 5	0.685**
Item 6	0.591**
Item 7	0.709**
Item 8	0.728**
Item 9	0.563**
Item 10	0.607**
Item 11	0.328**
Item 12	0.308**
Item 13	0.616**
Efficiency	
Item 1	0.669**
Item 2	0.236**
Item 3	0.281**
Item 4	0.631**
Item 5	0.642**
Item 6	0.778**
Item 7	0.809**
Item 8	0.755**
Item 9	0.700**
Cost of treatment	
Item 1	0.159**
Item 2	0.620**
Item 3	0.548**
Item 4	0.739**
Item 5	0.725**
Hospital selection	
Item 1	0.634**
Item 2	0.629**
Item 3	0.687**
Item 4	0.649**
Item 5	0.735**
Item 6	0.675**
Item 7	0.713**
Item 8	0.735**
Item 9	0.589**
Item 10	0.692**
Item 11	0.566**

**Table 2:** Domains-total correlation. **: correlation is significant at the level of 0.01 (2-tailed).

Domains	Total (r)
Services/facilities	0.831**
Efficiency	0.727**
Cost of treatment	0.195**
Hospital selection	0.610**

Cronbach’s alpha was used to assess reliability, with the coefficient values presented in Table 3. The overall Cronbach’s alpha coefficient was 0.869, indicating adequate instrument reliability.

**Table 3:** Cronbach’s alpha coefficients.

#	Domains	Number of items	Cronbach's alpha coefficient
1	Hospital selection	11	0.865
2	Services/facilities	13	0.830
3	Efficiency	9	0.799
4	Cost of treatment	5	0.618
	Overall subscales	38	0.869

## Results

Demographic background

Table 4 presents the demographic background, including nationality, gender, age, qualification, employment status, family income, and the number of family members. The majority of respondents were Saudi nationals (577, 96.2%), while 399 (66.5%) were female. Participants from various age groups were represented in the study.

**Table 4:** Socio-demographic details of the participants (n=600).

Variables	Number	Percentage
Nationality		
Saudi	577	96.2
Non-Saudi	23	3.8
Gender		
Male	201	33.5
Female	399	66.5
Age		
20<30 years	173	28.8
30<40 years	222	37.0
40<50 years	132	22.0
50<60 years	73	12.2
Qualification		
High school or below	71	11.8
Diploma	72	12.0
Bachelor’s degree	303	50.5
Master	107	17.8
PhD	47	7.8
Employment status		
Working	104	17.3
Not working	165	27.5
Public sector	256	42.7
Private sector	75	12.5
Marital status		
Married	361	60.2
Single	190	31.7
Divorce	41	6.8
Other	8	1.3
Family monthly income		
Less than 3000 SR	22	3.7
3000-5000 SR	52	8.7
5000-10000 SR	153	25.5
10000-20000 SR	241	40.2
20000 SR and above	132	22.0
Number of family members		
<4	163	27.2
4-7	364	60.7
7-12	65	10.8
>12	8	1.3

Descriptive statistics

The strongest factor influencing patients' preference between public and private hospitals is the hospital selection attributes (3.79±0.65). However, participants were neutral, i.e., not sure or did not give an exact response towards services provided by public and private hospitals and hospital efficiency. This is because of the factors influencing patients' preference between public and private hospitals in Riyadh, Saudi Arabia (2.68±0.65), (3.06±0.67) respectively. Finally, the descriptive statistics output revealed that the cost of treatment was not a factor influencing patients' preference between public and private hospitals (2.56±0.66).

Inferential statistics

An independent sample t-test was used to compare patients’ perceptions of factors influencing the preference of patients between public and private hospitals in Riyadh, Saudi Arabia, according to demographic variables. The results of the independent sample t-test are provided in Tables 5-7.

Those with a diploma or higher education (undergraduate, Master’s, or PhD) are more likely to consider private hospitals as providing a superior service relative to public hospitals (p-value<0.001) (Table 5). The finding highlights that more educated patients are better able to differentiate the level of service offered on the basis of factors such as the ease with which they can approach the staff or communicate with medical practitioners, the ability to easily book the services in private compared to public hospitals, cleanliness, waiting time and the level of customer service offered by staff to patients. Public hospitals are considered to offer an inferior service, especially to more educated patients whose expectation of the service level is unmet.

**Table 5:** ANOVA results for patients’ perception towards factors influencing the preference of patients between public and private hospitals according to education. *p<0.05, ** p<0.01.

Domain	Sum of squares	df	Mean square	F	p-value
Services provided					
Between groups	27.11	4	6.77	17.88	0.000**
Within groups	223.22	589	0.37		
Efficiency					
Between groups	4.16	4	1.04	2.34	0.054
Within groups	262.11	589	0.44		
Cost of treatment					
Between groups	5.98	4	1.49	3.52	0.007**
Within groups	250.24	589	0.42		
Hospital selection					
Between groups	1.85	4	0.46	1.11	0.348
Within groups	244.59	589	0.41		

Patients with low income were more likely to agree that the efficiency of hospitals was a factor that influenced their preference in choosing between public and private hospitals, compared to the patients with higher income (p-value=0.021) (Table 6). 

**Table 6:** ANOVA results for patients’ perception towards factors influencing the preference of patients between public and private hospitals according to monthly family income. *p<0.05, ** p<0.01.

Domain	Sum of squares	df	Mean square	F	p-value
Service provided					
Between groups	9.364	4	2.34	5.71	0.000**
Within groups	243.91	595	0.410		
Efficiency					
Between groups	5.20	4	1.301	2.92	0.021*
Within groups	264.80	595	0.445		
Cost of treatment					
Between groups	1.903	4	0.476	1.105	0.353
Within groups	256.22	595	0.431		
Hospital selection					
Between groups	3.20	4	0.800	1.892	0.110
Within groups	251.68	595	0.423		

Cost of treatment is a significant factor in influencing patient preference between private versus public hospitals in Riyadh based on the level of education (p-value=0.007) (Table 5). Non-Saudi patients were more likely to differentiate between public and private hospitals based on the cost of treatment. They were more likely to consider the cost of treatment to be higher in the private sector (p-value=0.043) (Table 7) but also acknowledge the more competitive service received (thus, private hospitals providing a better value for money proposition). They were also more likely to acknowledge the high indirect costs associated with public hospitals, such as the cost of appointments and travel.

**Table 7:** Patients’ perception towards factors influencing the preference of patients between public and private hospitals according to nationality. *p<0.05, ** p<0.01.

	Nationality	N	Mean	SD	t	p-value
Services provided	Saudi	577	2.67	0.65	1.337	0.182
	Non-Saudi	23	2.86	0.55		
Efficiency	Saudi	577	3.06	0.67	0.376	0.707
	Non-Saudi	23	3.01	0.66		
Cost of treatment	Saudi	577	2.55	0.65	2.033	0.043*
	Non-Saudi	23	2.83	0.69		
Hospital selection	Saudi	577	3.79	0.65	0.596	0.551
	Non-Saudi	23	3.71	0.64		

## Discussion

This work investigated the factors influencing the preference of patients between public and private hospitals in Riyadh, Saudi Arabia. Descriptive statistics and inferential statistics using independent sample t-tests helped to analyze the survey findings to corroborate and compare against the findings of the past literature. Most participants disagreed/strongly disagreed regarding the ease with which patients in public hospitals approach the reception staff when compared to the private hospitals (mean=2.51). The inferior service quality of public hospitals was also supported by the disagreement among research participants when asked if patients are able to secure easier online booking services in public hospitals compared to private hospitals (40% strongly disagreed, and 30% disagreed).

Student t-tests revealed interesting insights into the role of services in influencing patient preference between public and private hospitals in Riyadh. A comparison of the mean response of participants revealed that those with a diploma or higher education (undergraduate, Master’s, or PhD) were more likely to consider private hospitals as providing a superior service relative to the public hospitals (significant at 5% level). The finding highlights that more educated patients are better able to differentiate the level of service offered on the basis of factors such as the ease with which they can approach the staff or communicate with medical practitioners, the ability to easily book the services in private compared to public hospitals, cleanliness, waiting time and the level of customer service offered by staff to patients.

The findings of this study on the perception of service of public versus private hospitals in Riyadh can be corroborated by the findings of the literature review. Alumran et al. noted that employees in the private sector tend to be more professional, provide a differentiated and personalized service, are never too busy to take the time out and respond to requests from patients and their family members, and are more likely to provide individualized attention [1]. Those with higher education and exposure are better positioned to be able to differentiate between the service level of hospitals, which explains the ability of more educated (Diploma and above, as uncovered in this study) patients to hold a perception that they consider private hospitals in Saudi Arabia to possess superior facilities and quality of service [1]. 

The Student t-test output revealed that public hospitals are considered to offer an inferior service, especially to more educated patients whose expectation of the service level is not met. The challenges facing public hospitals (such as inferior service and doctors under pressure to treat more patients due to under-resourcing) could be explained by lower investment in facilities undertaken by the public hospitals in Riyadh. This is supported by the literature: greater investment within private relative to public hospitals is also reflected in the level of cleanliness and hygiene, as well as the quality of food provided [12]. This also answers why patients perceive private hospitals to provide a higher quality of service and possess superior facilities when compared to public hospitals.

On efficiency, patients with low income were more likely to agree that the efficiency of hospitals was a factor that influenced their preference in choosing between public and private hospitals, compared to the patients with higher income. For patients on lower income, they have a natural preference for public hospitals because they are less likely to afford private hospital costs [6]. Affordability influences the way low-income patients view their experience in public hospitals. For instance, patients on low income were more likely to view doctors treating patients in public hospitals in a friendly manner; they are more likely to hold a view that doctors provide complete answers to patients’ questions, they are free to ask questions, consider the nurses and other professional staff to be adequately qualified in public hospitals, doctors take a genuine interest. They feel confident in the competence of doctors in public hospitals [20]. 

The findings on the perception of the efficiency of public versus private hospitals in Riyadh (as uncovered through the t-test) can be corroborated by the findings of the literature. The perception of equal efficiency of public and private hospitals for those on low income is based on their limited experience in private hospitals because they can largely afford them and are likely to go to public hospitals [6]. High-income patients are likely to consider private hospitals in Riyadh to be more efficient. The relative inferior efficiency of public sector hospitals in Saudi Arabia has been reported by past studies [5]. Public hospitals have a non-profit objective. Therefore, they do not have the same incentives and drive, especially in the short run [7]. The increased inherent efficiency of the private hospitals relative to the public hospitals among those on high income (as reported in this study) means there is a strong economic rationale for promoting privatization of the healthcare sector as outlined by the Health Sector Transformation Program based on Kingdom’s Vision 2030 to address the challenges faced by healthcare sector in Saudi Arabia [13].

On the cost of treatment, 36% of respondents strongly disagreed, and 37% of the respondents disagreed that the indirect cost of obtaining an appointment with public hospitals pushes them to seek services from private hospitals in their area (mean=2.1). It indicates that indirect costs associated with public hospitals may not be a significant factor in encouraging the use of private hospitals in Riyadh. The finding was reinforced by another question in the survey. When asked about whether respondents experience spending more in the public hospital in Riyadh compared to the private hospital, 37% strongly disagreed, and 32% disagreed with this statement (mean=2.1). The finding is in contrast to the earlier conclusion of Al-Hanawi et al., who reported that even though public hospitals are more cost-effective and the direct cost of healthcare is less in public hospitals, the lower cost of healthcare services is often offset by the higher indirect costs levied on the patients when they visit public hospitals. For instance, indirect costs such as travel and unpaid leave needed for medical purposes (absence from work) were cited as two important costs [6]. The findings also refute the conclusion of Albejaidi et al. that the growing indirect cost of healthcare in public hospitals in Saudi Arabia is an important reason that incentivizes the growing number of patients to seek healthcare from private hospitals [16].

Respondents in this study agreed that the higher cost of private hospitals coincides with superior treatment, thereby justifying the higher cost due to better value for money. 30% of the respondents agreed, and 12% strongly agreed that the cost of treatment is higher in private hospitals, but the patients receive better treatment. An independent sample t-test revealed that the cost of treatment is a significant factor in influencing patient preference between private and public hospitals in Riyadh based on their nationality and level of education. Non-Saudi patients were more likely to differentiate between public and private hospitals based on the cost of treatment. Non-Saudi patients were more likely to consider the cost of treatment to be higher in the private sector but also acknowledge the more competitive service received (thus, private hospitals provide a better value for money proposition). They were also more likely to acknowledge the high indirect costs associated with public hospitals, such as the cost of appointments and travel. Non-Saudi patients are more likely to pay attention to the factors such as cost incurred (direct and indirect) because of anchoring bias: they are likely to compare the treatment cost incurred in Saudi Arabia to the cost they incurred in their home country or previous country of residence [21].

Finally, when it comes to hospital selection, most respondents also agreed or strongly agreed that the reputation of doctors employed by the hospitals, overall appearance and outlay of the hospital facilities, hygiene, and location of the hospitals are other variables that influence the choice of hospital among patients in Riyadh. One of the limitations of this study is the use of convenience sampling, which may limit the generalizability of the findings. Another limitation is the use of self-administered questionnaires, which may introduce the possibility of response bias. Also, distributing the survey electronically might have excluded individuals without internet access. Additionally, the cross-sectional design of the study may prevent the establishment of causal relationships between the identified factors and hospital preferences. The findings help to provide a number of recommendations to both the public and private hospitals in Saudi Arabia. Firstly, public hospitals should realize the significance of services and facilities in influencing the preference of patients when selecting a hospital. Facilities and services offered by public hospitals should be improved if they are to maintain and enhance the level of patient satisfaction and, eventually, the number of patient visits, which will enhance the quality of patient care provided by the public hospitals. There is a need to invest in the customer service training of staff including doctors at public hospitals, which will improve their ability to offer a more dedicated training. Secondly, the Saudi Arabian government should increase the budget of public sector hospitals where the ratio of patients per doctor is on the increase. A higher budget will allow hospitals to recruit more doctors and other support staff (such as nurses), enabling each doctor to allocate more time to their patients, as is the case in private hospitals.

Finally, the efficiency of public hospitals also needs to be enhanced. The efficiency improvements are premised on the ability of public hospitals to invest in modern equipment and technology. The Saudi Arabian government should allocate more funding to public sector hospitals, part of which should be dedicated to the investment in modern healthcare equipment and technology used in hospitals. Access to the latest equipment will improve the capital and labor productivity of workers while providing a more competitive and efficient level of service. The management of public hospitals should also introduce key performance indicators (KPIs) focused on hospital efficiency, which is used in private hospitals. Regular review of KPIs will provide a strong incentive to improve the efficiency of their operations.

## Conclusions

This study investigated patient preferences for public or private hospitals in Riyadh, Saudi Arabia. Higher-educated patients preferred private hospitals, and this was due to the provision of a superior service relative to public hospitals. Lower-income patients were more likely to agree that the efficiency of hospitals was a factor that influenced their preference. The lower cost of public hospitals remains an important attraction that encourages them to rely on public hospitals. Compared to Saudi patients, non-Saudi patients were more likely to differentiate between public and private hospitals based on the cost of treatment.
